# Comparison between surgical and percutaneous tracheostomy effects on procalcitonin kinetics in critically ill patients

**DOI:** 10.1186/s13054-018-2245-0

**Published:** 2018-11-14

**Authors:** Maria Vargas, Pasquale Buonanno, Lina Giorgiano, Giovanna Sorriento, Carmine Iacovazzo, Giuseppe Servillo

**Affiliations:** Department of Neurosciences, Reproductive and Odontostomatological Sciences, University of Naples “Federico II”, via pansini, Naples, Italy

**Keywords:** Tracheostomy, Procalcitonin, Infection, Sepsis, C-reactive protein, Critically ill patients

## Abstract

Available evidence from randomized controlled trials including adult critically ill patients tends to show that percutaneous dilatational tracheostomy (PDT) techniques are performed faster and reduce stoma inflammation and infection but are associated with increased technical difficulties compared with surgical tracheostomy (ST). A recent meta-analysis found that PDT was superior to reduce risk of periprocedural stoma inflammation and infection compared with ST. WE found no differences in procalcitonin, C-reactive protein, SOFA, and SAPS II between critically ill patients with ST or PDT.

In critically ill patients, tracheostomy may be performed with surgical or percutaneous approaches [1]. Available evidence from randomized controlled trials including adult critically ill patients tends to show that percutaneous dilatational tracheostomy (PDT) techniques are performed faster and reduce stoma inflammation and infection but are associated with increased technical difficulties compared with surgical tracheostomy (ST) [2, 3]. Overall complication rates are similar for PDT and ST, but with an increased incidence of infection for ST [4]. A recent meta-analysis found that PDT was superior to reduce risk of periprocedural stoma inflammation and infection compared with ST [4]. In the elderly population, fever is the most common postoperative complication after ST (42%), followed by wound infection (4%) [4]. Procalcitonin (PCT) may be a reliable biomarker to predict infectious or septic complications related to tracheostomy performed in the ICU [5]. A retrospective study reported that PCT was not elevated after ST performed in the ICU [5]. However, little is known about procalcitonin kinetics after ST or PDT in critically ill patients, since ST seems to be associated with an increased incidence of infection in this cohort of patients.

We screened 122 critically ill patients for tracheostomy, of which 12 received ST and 13 received PDT (Table 1). We found no difference in the baseline characteristics of patients between the two groups. Upper respiratory, blood, and urinary cultures performed 3 days before the procedure were negative for each patient. We found no difference between PCT, C-reactive protein (CRP), Sepsis Organ Failure Assessment (SOFA) score, and Simplified Acute Physiology Score (SAPS) II between the groups (all *p* > 0.05; Fig. 1). Upper respiratory, blood, and urinary cultures performed 3 days after the procedure were negative for each patient. The trends of PCT levels over time did not correlate with the trend of CRP levels in each group (ST group, *r* = 0.074, *p* = 0.671; r^2^ = 0.139, *p* = 0.425; PDT group, *r* = − 0.169, *p* = 0.297; r^2^ = − 0.063, *p* = 0.697).

**Fig. 1:**
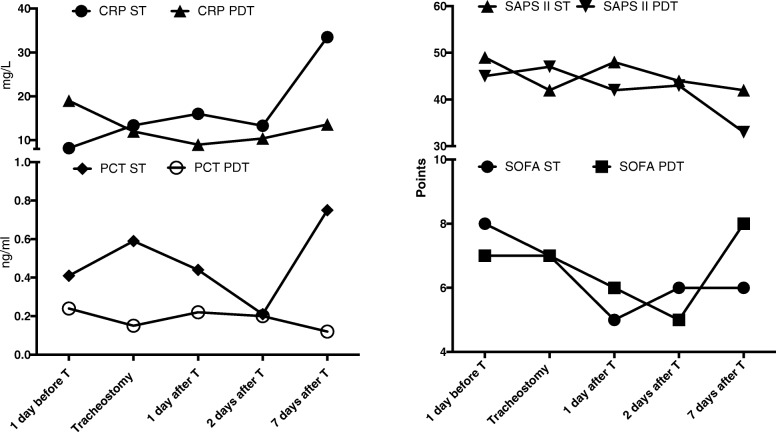
The PCT, CRP, SOFA, and SAPS II values remained stable over time for both groups (ST group, *p* value for PCT = 0.530, *p* value for CRP = 0.588, *p* value for SOFA = 0.480, *p* value for SAPS II = 0.289; PDT group, *p* value for PCT = 0.176, *p* value for CRP = 0.419, *p* value for SOFA = 0.402, *p* value for SAPS II = 0.993. *Left panel*: CRP and PCT kinetics in critically ill patients who underwent surgical and percutaneous tracheostomy. *Right panel*: SAPS II and SOFA scores in critically ill patients who underwent surgical and percutaneous tracheostomy. *CRP* C-reactive protein, *PCT* procalcitonin, *SOFA* Sepsis Organ Failure Assessment, *SAPS* Simplified Acute Physiology Score, *ST* surgical tracheostomy, *PDT* percutaneous dilatational tracheostomy, *T* tracheostomy

**Table 1:** Characteristics of included patients

	Surgical tracheostomy (*n* = 12)	Percutaneous dilatational tracheostomy (*n* = 13)	*p*
Age (years)	60 ± 10	56 ± 10	0.778
Gender (M/F)	8/5	7/6	0.539
BMI	20 ± 10	20.6 ± 8	0.345
Reason for ICU admission (N)			0.678
- Medical	7	6	
- Trauma	3	4	
- Surgical	2	3	
Duration of endotracheal intubation before T	15 ± 3	13 ± 5	0.257
Variables during procedure (N)			0.675
- Antibiotics	3	4	
- Corticosteroid	4	3	
- Fever (> 37°)	0	0	

To our knowledge this is the first report evaluating the kinetics of different biomarkers of infection in a cohort of tracheostomized patients. According to the literature, ST was associated with an increased risk of infections [4, 5]. We found that the biomarkers of infection were not different between the ST and PDT groups and remained stable in the first week after the procedure. According to these data, ST may not increase the risk of infections and sepsis in critically ill patients.

## Abbreviations

CRP: C-reactive protein
PCT: Procalcitonin
PDT: Percutaneous dilatational tracheostomy
SAPS II: Simplified Acute Physiology Score II
SOFA: Sepsis Organ Failure Assessment
ST: Surgical tracheostomy
